# SNHG5/miR-299-5p/ATF2 Axis as a Biomarker in Immune Microenvironment of Intervertebral Disc Degeneration

**DOI:** 10.1155/2022/2558275

**Published:** 2022-06-23

**Authors:** Yu Shi, Rong Guo, Yanyan Zeng, Qian Fang, Xianglong Wang, Wei Liu, Guozhi Huang, Wen Wu

**Affiliations:** ^1^Department of Rehabilitation, Zhujiang Hospital, Southern Medical University, Guangzhou 510282, China; ^2^Department of Rehabilitation, Guangzhou Red Cross Hospital, Jinan University, Guangzhou 510000, China

## Abstract

**Methods:**

The data sets of GSE56081 and GSE63492 in the Gene Expression Omnibus (GEO) database were used for screening and analysis, and the key gene markers were verified by GSE34095 and GSE126883. Finally, the infiltration of immune cells in the data were analyzed by MCPcounter analysis package.

**Results:**

In this study, a ceRNA containing 15 lncRNAs, 9 miRNAs, and 103 mRNAs was constructed. After multimodel screening and verification, key gene marker was found, namely, ATF2. The lncRNA/miRNA/mRNA axis closely related to ATF2 have also been found, namely, SNHG5/miR-299-5p/ATF2. In the analysis of immune infiltration, ATF2 was negatively correlated with T cells but positively correlated with neutrophils and endothelial cells.

**Conclusion:**

The SNHG5/miR-299-5p/ATF2 can be used as biomarker of IDD, and infiltration of immune cells plays an important role in the pathological development of IDD. In addition, as a marker of IDD, the involvement of the above-mentioned axis in the pathological development of IDD remains to be further explored.

## 1. Introduction

Back pain is one of the most common pain in modern society. It is estimated that 95% of people will experience back pain occasionally or for a long time at some point in their life [1]. Prolong back pain will not only bring serious economic burden to patients but also lead to serious mental stress and even suicidal tendencies [2]. Back pain is closely related to intervertebral disc degeneration (IDD) [3]. Due to the degeneration of intervertebral disc, there will be pathological changes such as sciatica, disc herniation/prolapse, and spinal stenosis, which will lead to back pain from cervical spine to tailbone [4]. Therefore, it is of great significance to understand the mechanism of IDD and to slow down the possible process of IDD for reducing back pain. At present, the diagnosis of IDD is still mainly based on the clinical manifestations and imaging information when the symptoms appear, and there is no great progress in the early diagnosis [5]. The absence of early diagnostic markers may affect early intervention in patients. Therefore, the search for early biological markers of IDD can not only better maintain the biological function of the patient's intervertebral disc but also reduce the probability of the possible occurrence of back pain, which has important clinical significance.

At present, the mechanism of IDD is still unclear, and researchers have proposed a variety of theories to explore the causes of its occurrence and development [6]. Some researchers have pointed out that the degeneration of the disc may be related to the physical force, and the longtime of daily stress causes the disc gradually “loss of water,” eventually leading to degeneration [7]. Other studies believe that IDD is closely related to hormone regulation, especially the decrease of estrogen level in menopausal women may lead to the occurrence of IDD [8]. In addition to the above studies, some researchers believe that the occurrence and development of IDD are closely related to immune cell infiltration. Studies have shown that IDD is closely related to the activation of T cells [9]. T cells can participate in the inflammatory response of IDD by highly expressing gamma interferon (IFN-*γ*) [10, 11]. At the same time, the IFN-*γ* also participates in the activation of macrophages in the inflammatory response of IDD and jointly affects the immune microenvironment of IDD [12]. In addition, there is evidence that the immune response involved in IDD may be related to the nucleus pulposus. Due to the structure of the disc, the nucleus pulposus in the center is isolated from the immune circulatory system [13]. At the same time, the nucleus pulposus cells release Fas ligand (FasL) tumor necrosis factor to destroy the infiltrating immune cells and maintain immune isolation [14, 15]. In IDD, nucleus pulpous cells will reduce the release of FasL, and nucleus pulpous cells will also be exposed to the immune circulation system, stimulating the immune system to release a large number of T cells and B cells, thus breaking the balance of the intervertebral disc immune microenvironment, and leading to the occurrence of inflammatory cascade reaction [16]. Therefore, it is of great significance to explore the role of IDD key markers in immune infiltration and the changes of immune microenvironment from the perspective of immune cell infiltration to reveal the mechanism of IDD. At the same time, it is of great value in clinical practice to actively explore the possible intervention pathways of IDD based on the above mechanisms. MCPCounter (MCP) is used to estimate the abundance of tissue-infiltrating immune cells and other stromal cells [17]. MCP was first used in the analysis of cancer-related immune infiltration [18], and now more and more researchers are applying it in other immune-related studies of nontumor inflammatory response [19].

In order to improve the reliability of research results, coding RNA and noncoding RNA data from the same sample set were analyzed and estimated from Gene Expression Omnibus (GEO) database in this study. Key marker genes were obtained through differential gene expression analysis, ceRNA network construction, random forest (RF) machine learning model screening, and receiver operating characteristic curve (ROC) verification. At the same time, the MCP immune infiltration database was used to analyze and summarize the degree of immune cell infiltration in the target data set, and correlation analysis was conducted in combination with the previously verified markers, so as to construct key biological markers that may affect the immune microenvironment of IDD, and point out the direction for further exploration of the immune mechanism of IDD.

## 2. Methods

### 2.1. Data Source

In this study, public data sets GSE56081 [20] and GSE63492 [21] in the GEO database were used as the training set for estimation and analysis, and data sets GSE34095 and GSE126883 [22] were used as the verification set to verify the results.

### 2.2. Data Preprocessing

The *R* (V4.0.4) software (https://www.r-project.org/) was used for data preprocessing, including missing value completion, correction, normalization, and other steps.

### 2.3. Analysis of Differentially Expressed Genes (DEGs)

The limma analysis package [23] was used to analyze the expression of lncRNAs, miRNAs, and mRNAs in the data set, so as to screen the DElncRNAs, DEmiRNAs, and DEmRNAs. Volcano maps were generated to show the differential expression of DEGs (DElncRNAs, DEmiRNAs, and DEmRNAs). DEGs with *p* < 0.05 and |log2FC| > 1 were considered statistically significant.

### 2.4. Screening for Intracellular Localization of lncRNA

The endogenous competitive effect of lncRNAs was mainly expressed in the cytoplasm. lncRNAs located only in the nucleus were excluded using the online database lncATLAS [24].

### 2.5. lncRNA Targeting Predicts miRNA

The starBase [25] online database was used to predict miRNAs that might bind to lncRNAs and intersected with DEmiRNAs to obtain hubmiRNAs, which were included in the next analysis.

### 2.6. miRNA Targeting Predicts mRNA

The starBase [25] and TargetScan [26] online databases were used to predict mRNAs that might bind to hubmiRNAs and intersected with DEmRNAs to obtain hubmRNAs, which were included in the next analysis.

### 2.7. Construction of ceRNA Network

The binding relationships among hublncRNAs, hubmiRNAs, and hubmRNAs analyzed in the above steps were used to construct the ceRNA network, which was presented by Cytoscape software [27].

### 2.8. Functional Enrichment Analysis

The online database DAVID (https://david.ncifcrf.gov) [28] was used for Gene Ontology (GO) enrichment analysis and Kyoto Encyclopedia of Genes and Genomes (KEGG) enrichment analysis of the hubmRNAs in ceRNA. The results with *p* < 0.05 were considered significant enrichment. The GOplot analysis package [29] was used to draw the GOCircle plot of GO enrichment analysis results, and the online website bioinformatics (http://www.bioinformatics.com.cn) was used to draw the GOBubble plot of KEGG enrichment analysis results.

### 2.9. Screening of RF Model

The random forest analysis package [30] was used to perform RF machine learning analysis on the data set to screen the key markers. The results of the analysis were intersected with the hubmRNAs to obtain the key gene markers. The online website Venny (https://bioinfogp.cnb.csic.es/tools/venny) was used to draw the Venn diagram.

### 2.10. Key Gene Marker Verification and ROC Verification

The bioinformatics was used to verify the expression of key gene makers in the verification datasets GSE34095 and GSE126883. At the same time, this dataset was used for ROC analysis and ROC curve was drawn. The results with *p* < 0.05 and AUC > 0.5 were considered statistically significant. Meanwhile, the ceRNA network was used to search for lncRNA~miRNA~mRNA axis related to key gene markers.

### 2.11. Correlation Analysis of lncRNA/miRNA/mRNA Axis

The corrplot analysis package [31] was used to analyze the correlation between the genes in the axis. The results with *p* < 0.05 were considered statistically significant.

### 2.12. Immune Infiltration Analysis

The MCPcounter analysis package was used to analyze and estimate the transcriptome matrix data included in this study, and the immune cell infiltration matrix data was obtained. The corrgram analysis package [32] was used to visualize the correlation between immune infiltrating cells involved in MCP.

### 2.13. Correlation Analysis between lncRNA/miRNA/mRNA Axis and Immune Infiltrating Cells

The tidyverse analysis package [33] and ggstatsplot analysis package (https://CRAN.R-project.org/package=ggstatsplot) were used to analyze the correlation between the genes of lncRNA/miRNA/mRNA axis and immune infiltrating cells. The bioinformatics was used to draw the correlation coefficient plot. The results with *p* < 0.05 were considered statistically significant.

## 3. Results

### 3.1. Results of Data Analysis Process

**Figure 1:**
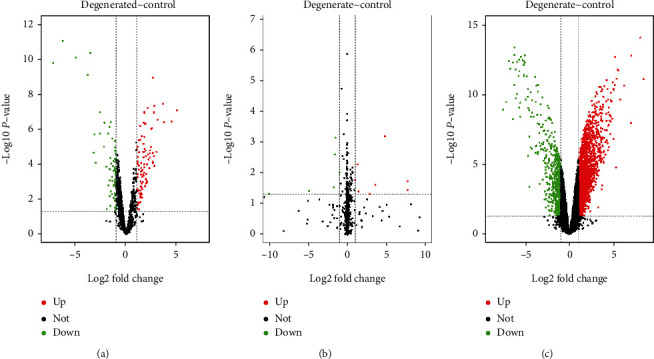
DEGs in the dataset. (a) Volcano map of DElncRNAs. Green represents downregulated DElncRNAs, and red represents upregulated DElncRNAs. (b) Volcano map of DEmiRNAs. Green represents downregulated DEmiRNAs, and red represents upregulated DEmiRNAs. (c) Volcano map of DEmRNAs. Green represents downregulated DEmRNAs, and red represents upregulated DEmRNAs.

**Figure 2:**
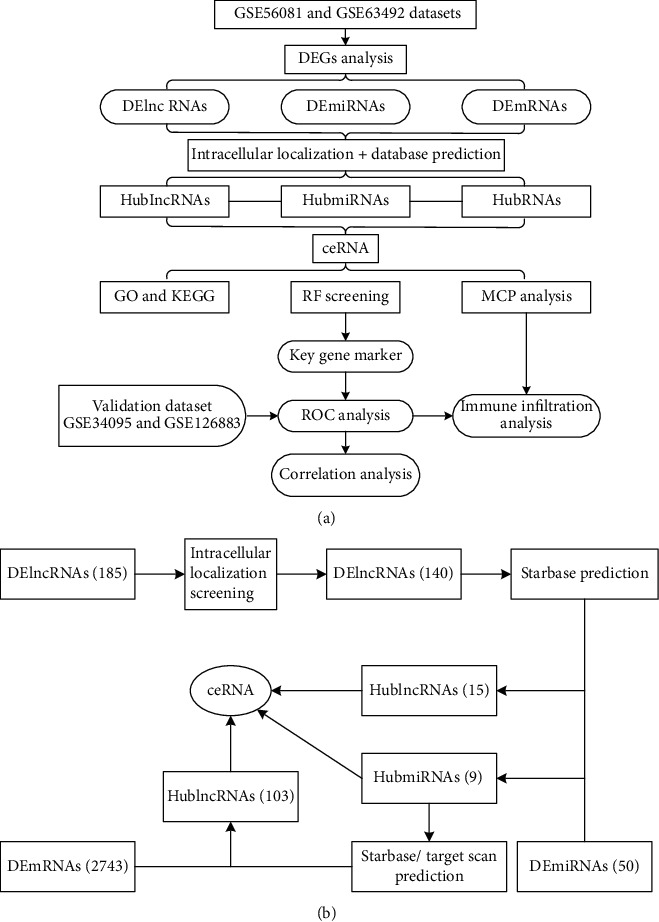
Flow chart of research analysis. (a) Whole process content of the study. (b) Process content of ceRNA network construction. DEGs: differentially expressed genes; GO: Gene Ontology; KEGG: Kyoto Encyclopedia of Genes and Genomes; RF: random forest; ROC: receiver operating characteristic curve.

In this study, GSE56081 and GSE63492 datasets were used for analysis. Firstly, a total of 185 DElncRNAs, 50 DEmiRNAs, and 2743 DEmRNAs were obtained after differential gene screening (Figure 1); secondly, after intracellular localization screening, 140 DElncRNAs remained; thirdly, a total of 15 hublncRNAs and 9 hubmiRNAs were found to be bound by online database prediction; fourthly, a total of 103 hublncRNAs and above 9 hubmiRNAs were found to be bound by online database prediction; finally, after RF model screening and ROC verification, a total of 1 key gene marker was obtained, namely, ATF2. At the same time, lncRNA/miRNA/mRNA axis closely related to key markers was also extracted, namely, SNHG5/miR-299-5p/ATF2, and included in the final analysis (Figure 2).

### 3.2. ceRNA Network

**Figure 3:**
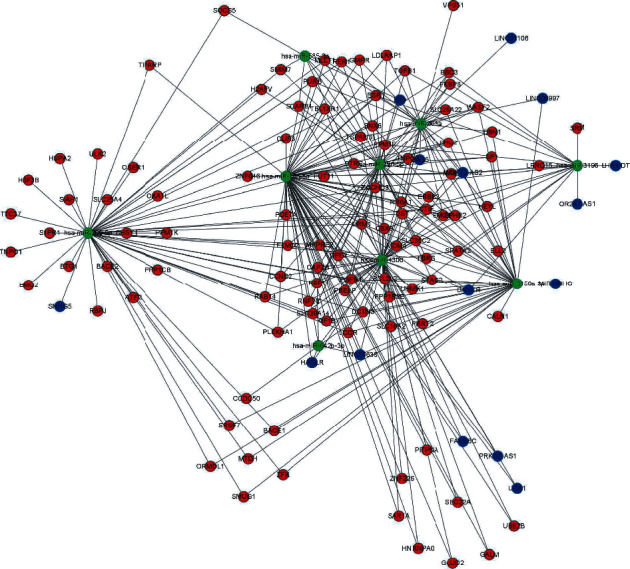
ceRNA network. The blue modules are lncRNA, the green modules are miRNA, and the red modules are mRNA.

A total of 15 hublncRNAs, 9 hubmiRNAs, and 103hubmRNAs were contained in the ceRNA network, including 289 binding relationships (Figure 3).

### 3.3. Results of GO and KEGG Enrichment Analysis

**Figure 4:**
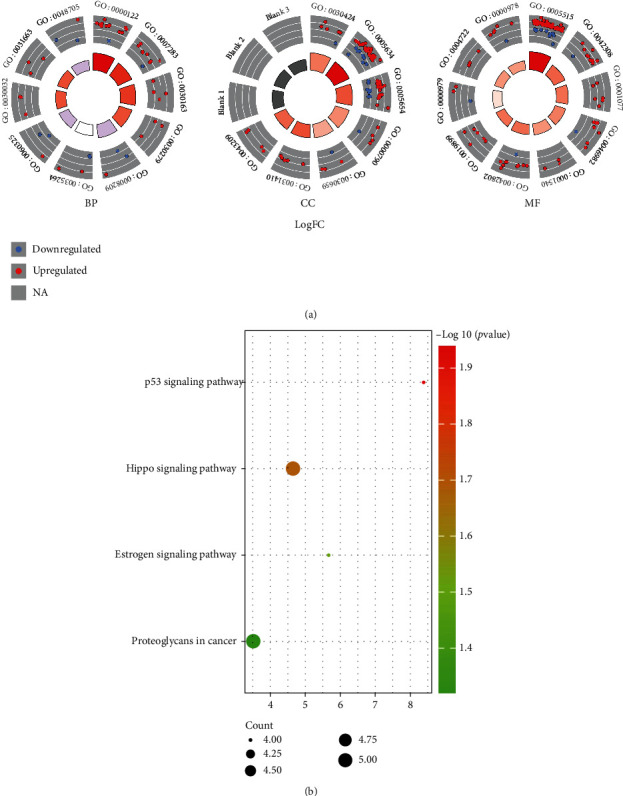
Results of GO and KEGG enrichment analysis. (a) GOCircle plot of GO enrichment analysis. (b) GOBubble plot of KEGG enrichment analysis.

The results of GO analysis showed that the hubmRNAs were mainly related to negative regulation of transcription from RNA polymerase II promoter, protein catabolic process, negative regulation of ossification, multicellular organism growth, lamellipodium assembly, lipopolysaccharide-mediated signaling pathway, skeletal system morphogenesis, cell migration, and positive regulation of protein phosphorylation in biological process (BP) (Figure 4(a)); mainly related to axon, nucleus, nucleoplasm, nuclear chromatin, cytoplasmic vesicle membrane, cytoplasmic vesicle, and myelin sheath in cellular component (CC) (Figure 4(a)); and mainly related to protein binding, protein homodimerization activity, protein heterodimerization activity, beta-amyloid binding, identical protein binding, and enzyme binding in molecular function (MF) (Figure 4(a)). These results suggest that hubmRNAs are mostly related to decomposition, synthesis, and transformation of protein and also participate in multiple signaling pathways. The results of KEGG analysis showed that hubmRNAs were mainly related to p53 signaling pathway, Hippo signaling pathway, estrogen signaling pathway, and proteoglycans in cancer (Figure 4(b)).

### 3.4. Results of RF Model Screening

**Figure 5:**
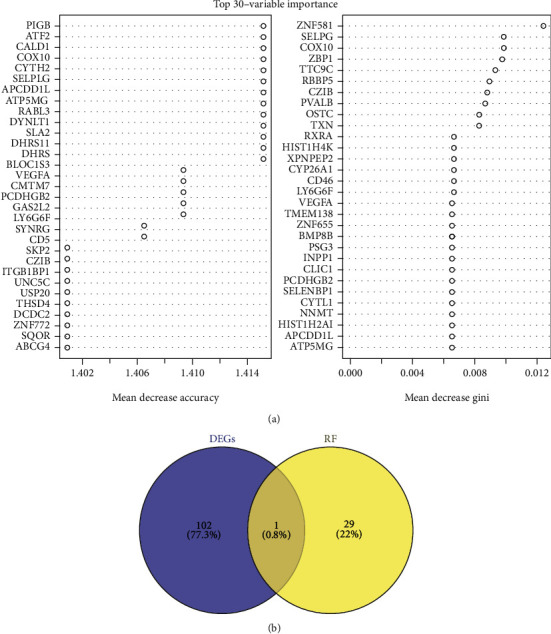
Results of RF model screening. (a) Top 30 genes obtained by RF model analysis. (b) Venn diagram of intersection between hubmRNAs and RF top 30 genes.

A total of 30 top genes were obtained by RF model analysis (Figure 5(a)), and a key gene marker was obtained after intersecting with hubmRNAs, namely, ATF2 (Figure 5(b)).

### 3.5. Results of Key Gene Marker Verification and ROC Verification

**Figure 6:**
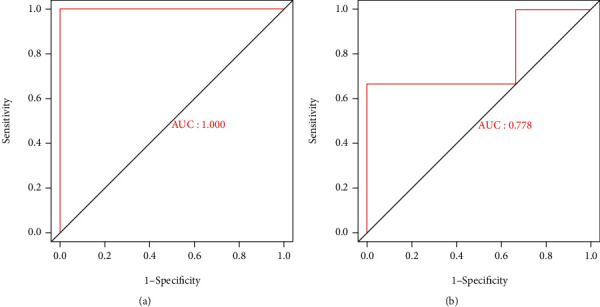
Results of ROC verification. (a) ROC curve of ATF2 in verification data set of GSE34095. (b) ROC curve of ATF2 in verification data set of GSE126883.

The ROC analysis results showed that ATF2 had good predictability in GSE34095 and GSE126883 (*p* < 0.05) (Figure 6).

### 3.6. lncRNA/miRNA/mRNA Axis Closely Related to Key Gene Marker

**Figure 7:**
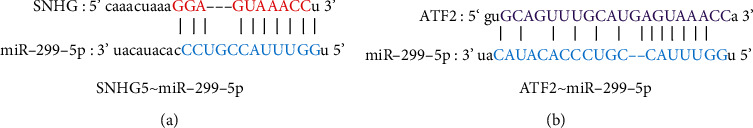
The binding site of genes. (a) The binding site of SNHG5/miR-299-5p. (b) The binding site of miR-299-5p/ATF2.

The lncRNA/miRNA/mRNA axis closely related to ATF2 was extracted from ceRNA, namely, SNHG5/miR-299-5p/ATF2. The figure shows the binding site of SNHG5/miR-299-5p and miR-299-5p/ATF2 (Figure 7).

### 3.7. Correlation Analysis Results of lncRNA/miRNA/mRNA Axis

**Figure 8:**
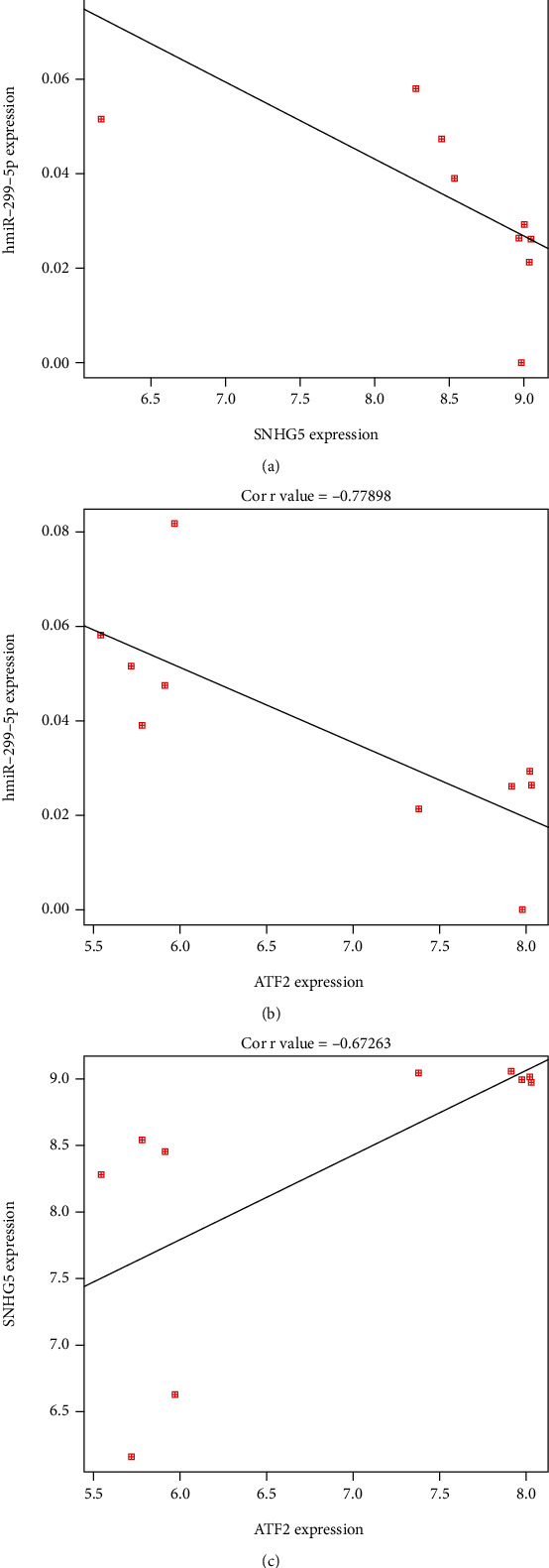
Correlation analysis results of genes. (a) Negative correlation between SNHG5 and miR-299-5p. (b) Negative correlation between ATF2 and miR-299-5p. (c) Positive correlation between SNHG5 and ATF2.

After correlation analysis, the results showed that SNHG5 and ATF2 were negatively correlated with miR-299-5p, as shown in Figures 8(a) and 8(b), while there was a positive correlation between SNHG5 and ATF2, as shown in Figure 8(c).

### 3.8. Correlation Analysis Results between Genes and Immune Infiltrating Cells

**Figure 9:**
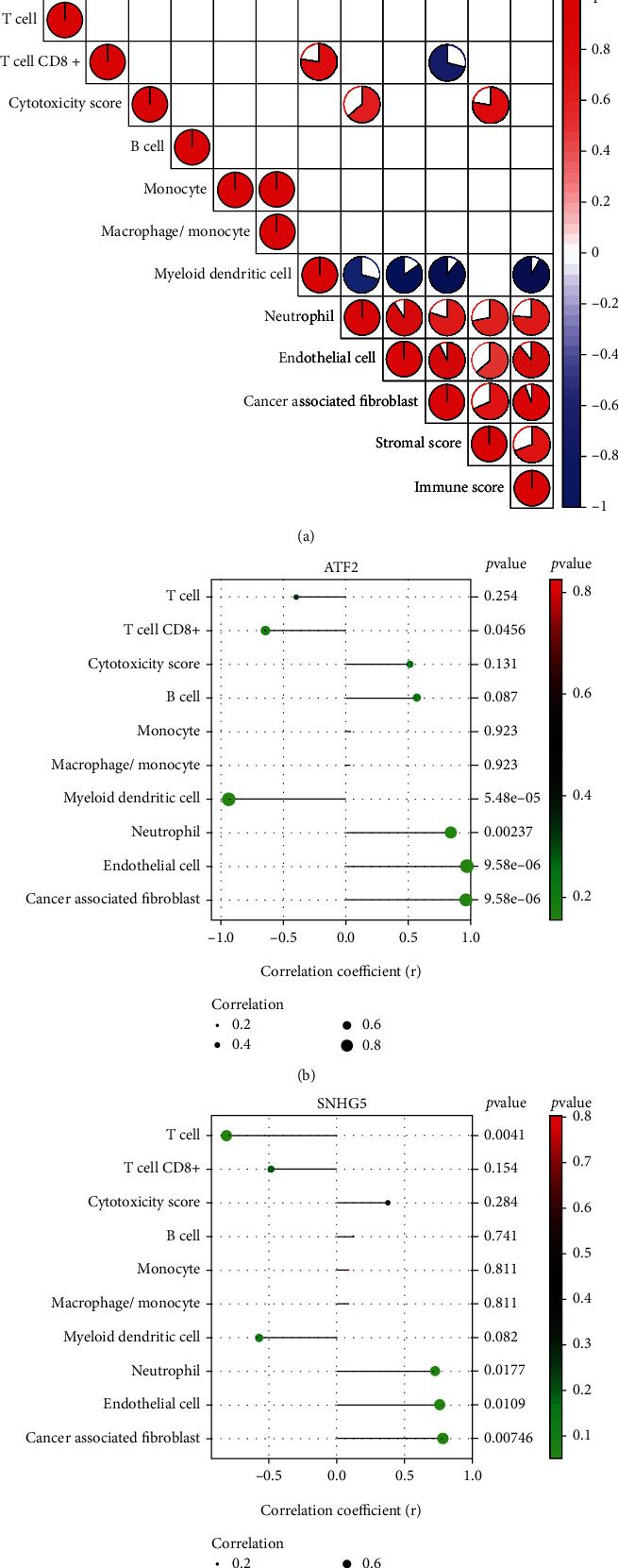
Correlation analysis results between genes and immune infiltrating cells. (a) Correlation analysis of immune infiltrating cells. (b) Correlation coefficient plot of ATF2 and immune infiltrating cells. (c) Correlation coefficient plot of SNHG5 and immune infiltrating cells.

Correlation analysis results showed that T cell CD8+ was positively correlated with myeloid dendritic cell but was negatively correlated with cancer-associated fibroblast. Monocyte was positively correlated with macrophage/monocyte; myeloid dendritic cell was negatively correlated with neutrophil, endothelial cell, and cancer-associated fibroblast; neutrophil was positively correlated with endothelial cell and cancer-associated fibroblast; endothelial cell was positively correlated with cancer-associated fibroblast (Figure 9(a)). ATF2 was positively correlated with neutrophil (*r* = 0.84, *p* < 0.01), endothelial cell (*r* = 0.966, *p* < 0.01), and cancer-associated fibroblast (*r* = 0.961, *p* < 0.01) but was negatively correlated with T cell CD8+ (*r* = −0.641, *p* = 0.0456) and myeloid dendritic cell (*r* = −0.939, *p* < 0.01) (Figure 9(b)); SNHG5 was positively correlated with neutrophil (*r* = 0.725, *p* = 0.0177), endothelial cell (*r* = 0.759, *p* = 0.0109), and cancer-associated fibroblast (*r* = 0.782, *p* < 0.01) but was negatively correlated with T cell (*r* = −0.815, *p* < 0.01) (Figure 9(c)).

## 4. Discussion

IDD is the aging process of intervertebral disc with age or other factors, which is closely related to back pain [34]. Finding the key markers of early IDD can effectively intervene the process of IDD and provide strong support for maintaining the biological function of intervertebral disc and preventing various complications. In this study, we found ATF2 as a key gene marker of IDD and also found an lncRNA/miRNA/mRNA axis closely related to ATF2, namely, SNHG5/miR-299-5p/ATF2. We found that above genes were closely related to immune infiltrating cells, suggesting that the lncRNA/miRNA/mRNA axis may affect the course of IDD by interfering with the immune microenvironment.

In the GO enrichment analysis results, we found that hubgenes were mainly enriched in the processes of production, phosphorylation modification, isomer formation, binding, and catabolism in protein. These results indicate that the occurrence of IDD is closely related to metabolic abnormalities. Studies have shown that the main biological change of IDD is the massive loss of proteoglycans, especially small molecules of proteoglycans. In IDD, proteoglycans are continuously degraded, and small molecules of proteoglycans are constantly exuding from the tissues, resulting in a decrease in osmotic pressure and continuous “loss of water” of the intervertebral disc [35]. Type II collagen has also been found to be more susceptible to denaturation and breakage in its helical structure in IDD, which affects the type and distribution of collagen in IDD [36]. In addition, the content of fibronectin increases as protein denaturation increases and becomes more fragmented [37]. The above research conclusions indicate that IDD will have obvious metabolic abnormalities of proteins and protein conjugates, which play a role in the occurrence and development of IDD. Our research results confirm the above research viewpoints. Our results also found that the hubgenes of IDD are also enriched in biological processes such as negative regulation of ossification, skeletal system morphogenesis, and cell migration. Intervertebral disc degeneration is an important cause of intervertebral disc calcification. As the intervertebral discs continue to age, bone spurs, fibrosis, and calcification appear in the intervertebral discs, causing spinal stenosis and nerve root compression [38]. Our results suggest abnormal protein metabolism and ossification in IDD. Meanwhile, we found several miRNAs in the ceRNA network, which may be involved in the IDD process. The role of some miRNAs has been confirmed in IDD studies. It has been proved that circ-TIMP2 promoted TNF-*α*- and IL-1*β*-induced degenerative nucleus pulposus cell imbalance between enhanced extracellular matrix anabolism and catabolism via miR-185-5p-MMP2 signaling [39]. Wang et al. suggested that circ-RERE promotes the hydrogen peroxide-induced apoptosis and autophagy of nucleus pulposus cells through the miR-299-5p/galectin-3 axis [40].

After multiple models screening, we obtained the SNHG5/miR-299-5p/ATF2 axis. We found that ATF2 and SNHG5 have low expression in IDD, and they had a positive correlation. And miR-299-5p was negatively correlated with the above two, suggesting the binding regulation relationship of miR-299-5p on SNHG5 and ATF2. ATF2 mRNA is responsible for encoding Activating transcription factor 2 [41]. In the cell cycle, ATF2 mainly combines with cyclin D1 to promote the proliferation and differentiation of chondrocytes. This process is regulated by cytokines such as transforming growth factor beta (TGF-*β*) and parathyroid hormone–related peptide (PTHrP) [42]. In Kashin-Beck disease, increased phosphorylation of ATF2 is associated with chondrocyte apoptosis [43], consistent with the previously defined role in osteoclast differentiation [44]. In this study, our results showed that ATF2 was underexpressed in IDD, and was negatively correlated with miR-299-5p, suggesting that ATF2-mediated chondrocyte proliferation decreased in IDD, and it may be affected by miRNA binding. ATF2 has also been shown to be closely related to macrophages and dendritic cells. ATF2 will stimulate these cells to release cytokines such as interleukin 23 (IL-23) through the JNK pathway and play a role in immune infiltration [45]. However, no such association was found in our study, suggesting that the low expression of ATF2 may not affect macrophages and dendritic cells, reflecting a characteristic of the immune mechanism of IDD. ATF2 will combine with c-Jun to form a heterodimer. After being stimulated by tumor necrosis factor alpha, it quickly completes phosphorylation and promotes the expression of E-selectin [46]. E-selectin is a key protein that promotes the migration of neutrophils to endothelial cells and a key molecule that regulates the process of endothelial-leukocyte adhesion [46]. In this study, we found that ATF2 is highly correlated with neutrophils and endothelial cells, suggesting that leukocyte-endothelial adhesion also appears in the IDD immune response, reflecting a characteristic of the changes in the IDD immune microenvironment. ATF2 mainly participates in the inflammatory response through the JNK and p38 MAP kinase pathways [47]. The low expression of ATF2 can lead to the excessive activation of the immune system and the massive invasion of T cells [11]. Our results found that the expression of ATF2 was negatively correlated with the content of T cells, suggesting the excessive activation of immune responses in IDD and the occurrence of immune cascades.

lncRNA is involved in many biological processes, such as cell migration, proliferation, cycle, apoptosis, and autophagy [48]. SNHG5 is closely related to the proliferation of collagen cells, participates in the miR-26a/SOX2 signal axis, and promotes the proliferation and migration of chondrocytes [49]. In this study, we found that the low expression of SNHG5 in IDD will affect the regeneration and repair of cartilage cells in IDD. SNHG5 can also regulate the miR-205-5p/ZEB2 axis to promote the proliferation of glial cells [50]. In the course of IDD, not only the aging and dehydration of intervertebral discs but also the degeneration and necrosis of the nervous system may occur. SNHG5 may affect the occurrence of neuralgia in IDD through the abovementioned pathways. SNHG5 participates in inflammatory response and cell apoptosis. It mainly regulates the abovementioned biological processes by promoting the molecule miR-155, which affects the release of a large number of inflammatory cytokines from endothelial cells, leading to the occurrence of cell apoptosis [51]. Our results found that SNHG5 is positively correlated with neutrophils and endothelial cells, suggesting that SNHG5 may play a role in the immune response of IDD through the above pathways. SNHG5 is also involved in the process of IL-1*β*-induced chondrocyte apoptosis, and it has been confirmed that SNHG5 impedes IL-1*β*-induced chondrocyte apoptosis by sponging miR-10a-5p [52]. In IDD, the antagonistic effect of this process is greatly reduced due to the low expression of SNHG5, which induces a large number of apoptosis of chondrocytes and plays a role in the aging of intervertebral disc. Previous studies have shown the role of miR-299-5p/ATF2 axis in immune escape of tumor cells. The low expression of ATF2 is closely related to the overactivation of the immune system, suggesting the mutual regulation between the immune microenvironment of IDD and miR-299-5p/ATF2 axis. ATF2 and SNHG5 showed a certain functional consistency in our research results. Both of them were positively correlated with neutrophils and endothelial cells but negatively correlated with T cells, suggesting the synergy of the two elements in the IDD immune mechanism and the possible unknown mutual regulation relationship. At present, the mutual regulation relationship between SNHG5/miR-299-5p/ATF2 and T cells needs to be clarified. Activated T cells are believed to be closely related to the repair of the nucleus pulposus in the early stage of IDD. However, excessive release of cytokines and inflammatory factors by T cells and other immune cells can cause the degeneration of the intervertebral disc to aggravate [16].

## 5. Limitations

Due to the limitation of sample size in the data used in this study, support vector machine (SVM) and least absolute shrinkage and selection operator (LASSO) models were not used for analysis in screening, which is a limitation in the study design. In addition, although multiple databases have been searched, the sample size of the verification data sets included in the current study is still relatively small, which is also a limitation of our study.

## 6. Conclusion

In this study, we found that the SNHG5/miR-299-5p/ATF2 can be used as biomarker of IDD, and infiltration of immune cells plays an important role in the pathological development of IDD. In addition, as a marker of IDD, the involvement of the above-mentioned axis in the pathological development of IDD remains to be further explored.

## Data Availability

The data set used in this study has been publicly shared on GEO (http://www.ncbi.nlm.nih.gov/geo/; accession numbers: GSE56081, GSE63492, GSE34095, and GSE126883).
